# Seasonal variation in bull semen quality demonstrates there are heat-sensitive and heat-tolerant bulls

**DOI:** 10.1038/s41598-022-17708-9

**Published:** 2022-09-12

**Authors:** Jacob K. Netherton, Benjamin R. Robinson, Rachel A. Ogle, Allan Gunn, Ana Izabel S. Balbin Villaverde, Kim Colyvas, Ced Wise, Tylah Russo, Amiee Dowdell, Mark A. Baker

**Affiliations:** 1 College of Heath, Medicine and Wellbeing, School of Biomedical Sciences and Pharmacy, University of Newcastle, University Drive, Callaghan, NSW 2308 Australia; 2 School of Agricultural, Environmental and Veterinary Sciences, Charles Sturt University, Wagga Wagga, NSW 2678 Australia; 3 Graham Centre for Agricultural Innovation (NSW DPI and CSU), Pugsley Place, Wagga Wagga, NSW Australia; 4 Institute of Biological and Natural Sciences, Federal University of Triângulo Mineiro, Uberaba, Minas Brazil; 5 College of Engineering, Science and Environment, University of Newcastle, Callaghan, NSW 2308 Australia; 6 Rocky Repro, Benjami 89 O’Brien Rd, Alton Downs, QLD 4702 Australia

**Keywords:** Biodiversity, Population dynamics, Ecology, Environmental sciences

## Abstract

Using semen data from 1271 ejaculates (79 different bulls, 11 different breeds) we have investigated the variability of semen quality in cattle living in sub-tropical conditions. Modelling shows definitive evidence of seasonal variation. Semen quality from the same bulls had a 90% “pass rate” for cryopreservation purposes in winter, dropping to less than 50% in summer. Notably, individual bulls could be classified as either “heat-tolerant” (produce good quality spermatozoa all year regardless of temperature) or “heat-sensitive” (only produce good quality sperm in summer). Nominal logistic regression demonstrated when temperatures reach 30.5 °C, 40% of heat-sensitive bulls fail a semen analysis 17 days later. At 34 °C, the proportion of bulls failing reached 63%. Ratifying this, the purposeful heating of bulls to 40 °C for 12 h showed that individual animals had different degrees of heat-sensitivity. Using historical temperature data, we then modelled how many days/decade bulls would be subject to heat-events. Beginning from 1939 to 1949, on average, the area in which bulls were kept recorded 19, 7 and 1 day over 38 °C, 39 °C and 40 °C respectively. This number steadily increases and of last decade (2010–2010), the numbers of days per decade over 38 °C, 39 °C and 40 °C jumped to a staggering 75, 39 and 15 respectively. These data show the urgent need to identify heat-tolerant bulls as future sires. Such variation likely explains why the veterinary bull breeding test often fails to accurately predict bull breeding potential.

## Introduction

To determine the fertility potential of a bull, a qualified veterinarian performs a veterinary Bull Breeding Soundness Evaluation (vBBSE). The vBBSE assesses the animal, including measurement of scrotal circumference, sperm motility and sperm morphology. The values obtained are then compared to a “normal” range of parameters and a prediction of the in vivo fertilisation potential of the bull is given. Although correlations between the vBBSE test and calf output have been reported for 70 years^1–4^ it is clear that the ability of the vBBSE to predict actual fertilisation rates is highly inconsistent. For example, collection of semen from 12 Holstein bulls (ranging between 6 and 11 years old) demonstrated that total sperm motility had a very poor predictive value to actual conception rates (r^2^ = 0.34). When progressive motility was measured, the predictive value increased (r^2^ = 0.68)^5^, which could be further improved by combining at least 5 different measurements of motility (lateral head displacement, beat-cross frequency, linearity, path velocity, straight line velocity). However, analysis of data from 117 young artificially inseminated bulls with at least 100 services each showed sperm motility was an extremely poor predictor of fertility with an r^2^ value between 0.13–0.24^6^. Correlations between bull fertility and sperm morphology^7–9^, or cell viability have also been performed and ranged from 0.06–0.86 and 0.33–0.66, respectively^7–9^. Although these correlations represent an improvement over fertility rates, they are by no means definitive. In Australia, the “bull power project” was established, consisting of 1000 bulls in the northern herds with the aim to correlate the vBBSE with calf output in higher numbers of animals. However, when “% normal morphology” was correlated to calf output, their conclusion was the same as Johnson reported^10^ in that there was no satisfactory relationship between bull fertility and sperm morphology^11^.

One issues with correlating sperm quality to fertility is studies performed in the 1940’s have shown that some bulls demonstrate a great variation from one ejaculate to the next^12^. In 1978, the source of the variation was investigated using 5033 ejaculates from 55 mature Holstein bulls. From this analysis, there was a modest increase (9%) in total sperm from the first ejaculate compared to the second. However, seasonal variation in semen quality was shown, with winter months being the lowest compared to summer (3.4–3.7 × 10^9^ vs 4.2 × 10^9^ total sperm). Further work demonstrating the effect of season and bull sperm quality has led to largely inconsistent conclusions. For example, whilst some have shown no seasonal effect on sperm motilty^13–17^, others have shown an increase during summer^18,19^ or (in complete contrast) winter^20–22^. Similarly, results have been obtained with total sperm production and sperm morphology with some reports suggesting season has no effect^13–15,23^ whilst others show an increase during summer^15,18,19,24^, or winter^13,16,17,23^. As such, opinions on whether seasonal alterations are indeed a source of variation in cattle semen quality are still divided. However, it is important to highlight that a closer examination of these studies shows that much of the contradictory results arise from the fact that these experiments were performed at very different locations. For example, in both Kenya and Thailand in which ambient temperature is consistent thoughout the year, little variation in semen quality was observed^13,17^. However, in USA^14^ and Sweden^20^ where winter temperatures drop below zero, it is little surprise that sperm motilty is higher during summer.

In addition to season, semen quality may change as a result of bull age, nutritional status and breed^14,25,26^. Therefore, the purpose of this study was to understand what are the major sources of semen variation of bulls maintained in the sub-tropical climate and explain why the vBBSE has poor predctive abililty. This knowledge will guide the proposal of practical measures, allowing an easier identification of subfertile bulls (e.g., changes to the vBBSE recommendations) and the improve in fertility, which will ultimately result in decreased food costs.

## Materials and methods

All chemicals were purchased from Sigma-Aldrich (Castle Hill, NSW, Australia) unless otherwise stated at the highest research grade.

### Preparation of bull spermatozoa and ethics

Institutional and State Government ethical approval was obtained for the use of bulls involved in this research programme through the University of Newcastle Animal Ethics committee (A-2020-008). All methods were carried out in accordance with the approved guidelines and reported according to the ARRIVE guidelines (arriveguidlines.org). All bulls were allowed to fed ad libitum in a paddock, and their diet was supplemented with a mixed ration [68.4% (wt/wt) dry matter, 7.2% (wt/wt) protein, 36.2% (wt/wt) neutral detergent fibre, 20.0% (wt/wt) acid detergent fibre, 6.07 net energy MJ, and 3.5 g minerals/kg (NaCl, Ca, and P) on a dry matter basis]. The semen samples were collected by electroejaculation no more than twice per week. Samples were collected into a warmed (38 °C) sterile 10 mL centrifuge tube and immediately placed into an incubator set to 38 °C. As the bulls were being used for stud purposes, often a second sample (recorded in supplementary 1) was taken. An aliquot of cells was taken and viewed under DIC microscope to assess sperm motility. A further 10 µL of sample was fixed in 1.0 mL 10% formalin for sperm morphology analysis.

### Climate conditions

The climate of the region where the study was conducted is considered sub-tropical. The climate data for each day from 01 Feb 2014 to 12 Aug 2019 was accessed from the Bureau of Meteorology and is available in Supplementary 2. Monthly average temperatures exceed 30˚C from October to March, then dropped to 23 °C in July. The relative humidity all year around generally sat between 80 to 100%, with very few days dropping below this value.

### Analysis of semen samples

Animals were left to acclimatise for a month at Rockhampton. The bull was restrained inside a cattle crush and semen was collected by electroejaculation into a warned 35 °C 15 mL plastic vial. Once collected, the sample was immediately placed into a 35 °C water bath before further assessment. Sperm motility was subjectively assessed by extending the semen approximately 1:1 with PBS and then scored at 100-400X magnification.

For morphology, 50 L of raw semen is added to 1–1.5 mL buffered formal saline (BFS). An aliquot was then viewed via DIC at 1500X magnification, and 100 cells were assessed for morphological characteristics. The sperm morphology traits were individually classified into eight different categories. Bulls that presented less than 70% of normal sperm forms, or at least one value below the threshold established for each morphological category, were considered “fail” for the morphology test. Abnormalities not to exceed: proximal cytoplasmic droplets (20%), vacuoles/teratoid (20%), knobbed acrosomes (30%), pyriform heads (20%), midpiece abnormalities (30%), swollen acrosomes (30%) and loose tails and heads (30%).

### Procedure for heat stress of bulls

A pre-existing shed was modified by fabricating an internal insulated wooden structure within the shed. The heating unit was built to 20 m × 3 m × 3 m using plywood. A back entrance gate was used to move the bulls in and out from the heating unit. After the plywood framework was built, the sides and roof were then insulated using R3.5 grade wool. The insulation was then covered with a black plastic wrap to allow better control over the air circulation. Two pedestal fans were installed on the roof heating and set to 2100 RPM in order to allow for air circulation within the heating barn. For added insulation, and to maintain 40 °C, the ground was covered with a thick 300 mm layer of straw. Eight electric thermometers were placed in even distribution around the barn. Two were placed close to the position of the testis of an average size bull in a standing position. The remaining six were placed in 500 mm increments above or below the latter, allowing the measure of a temperature gradient from the testis to the head of bulls. The temperature measured at the testis site was used as a guide to maintain the environmental temperature at 40 °C. It should be noted that despite fans circulating the air above, a 2 °C temperature gradient from head to testis (42–40 °C) was unavoidable.

In order to heat the room, a 21 kW electric blower heater, capable of airspeeds up to 230 L/sec, was used (Hired from Activeair.com.au). The unit was placed at one end of the shed and ducts were used to direct the air evenly around the heating barn. We conducted a series of tests over the course of three days to determine the appropriate settings and to calibrate the heating unit, thus, ensuring the barn could be heated and maintained at a constant temperature of 40 °C. Temperature probes were constantly monitored throughout the 12 h experiment, either directly or via a Bluetooth connection. To regulate the temperature, we found it necessary to have an opening (0.5 m diameter) near one of the duct entrances to the heating barn. As the daytime temperature rose, more air from the outside was allowed in, which maintained a constant 40 °C temperature within the building. Throughout the 12 h experiment, fluctuations of around 1–1.5 °C occurred. However, by regulating the heating unit air flow and/or by changing the opening diameter, to allow outside air to flow in, these temperature fluctuations never exceeded 3 min.

On the day of heating, the barn was heated for 2 h to equilibrate the room to 40 °C. The animals were housed inside the shed, but outside of the heating barn, which was a separate room within the shed. After 2 h, the bulls were introduced into the heating room. Within 10 min, the heating barn had returned to a temperature of 40 °C at testicular height, and the 12 h heating period commenced. Throughout the course of the work, the animals had access to water and straw *ad-libitum*. After 12 h of heating at 40 °C, the heating unit was turned off and the animals stayed in the heating barn for one hour to prevent cold shock. After this, they were released from the heating unit, but kept under the shed, with food and water *ad-libitum*. The next morning, the animals were released to their paddock.

### Statistical analysis

Bulls were classified into two groups, the first group being heat-insensitive if the percentage of a bull’s sperm samples that met or exceeded the normal sperm forms pass test (70% or more normal forms) was better than 80%. If not, then a bull was classified as heat-sensitive and only these bulls were used for further analysis. Pearson correlation coefficients were determined between sperm quality % and Tmax or THI for the day the sample was taken (lag 0) and up to lag 40, i.e. up to 40 days prior to the day the sample was taken. The correlations were also determined with averages of temperatures or THI over a number of days. For example, when 2 days were grouped the Tmax or THI values at lag 0 and lag 1 were averaged and correlated with sperm quality % on the day of the sample. The next lag was the average of lag 1 and lag 2 and so on until up to 40 days of history were used. The resultant set of correlation coefficients were plotted on the y axis against the day of the first lag in the group on the x axis. Groupings for the averages were 2, 3, 4 and 5 days. The lag with the largest (negative) correlation was chosen as indicating the point in time when the impact of Tmax or THI on sperm quality most likely took place.

An alternative approach was carried out on a subset of 12 bulls in the heat-sensitive group where each had at least a moderate amount of data (8–44 sperm samples and appeared to display a clear heat-sensitive effect. The analysis was based on converting sperm quality % into 3 categories, passed (sperm quality 70% or better), a qualified pass (50% or better if pass had failed) or failed (less than 50%) as these are criteria for spermiogram assessment for cryopreservation. A nominal logistic regression model was fitted to model the proportion of pass and qualified pass as a function of Tmax 17 days prior, the failed category being the reference condition, The predictions from the model were used to prepare a plot of proportion of sperm samples meeting the pass or qualified pass conditions as a function of Tmax 17 days prior.

## Results

### Intra-bull semen quality variation

**Figure 1:**
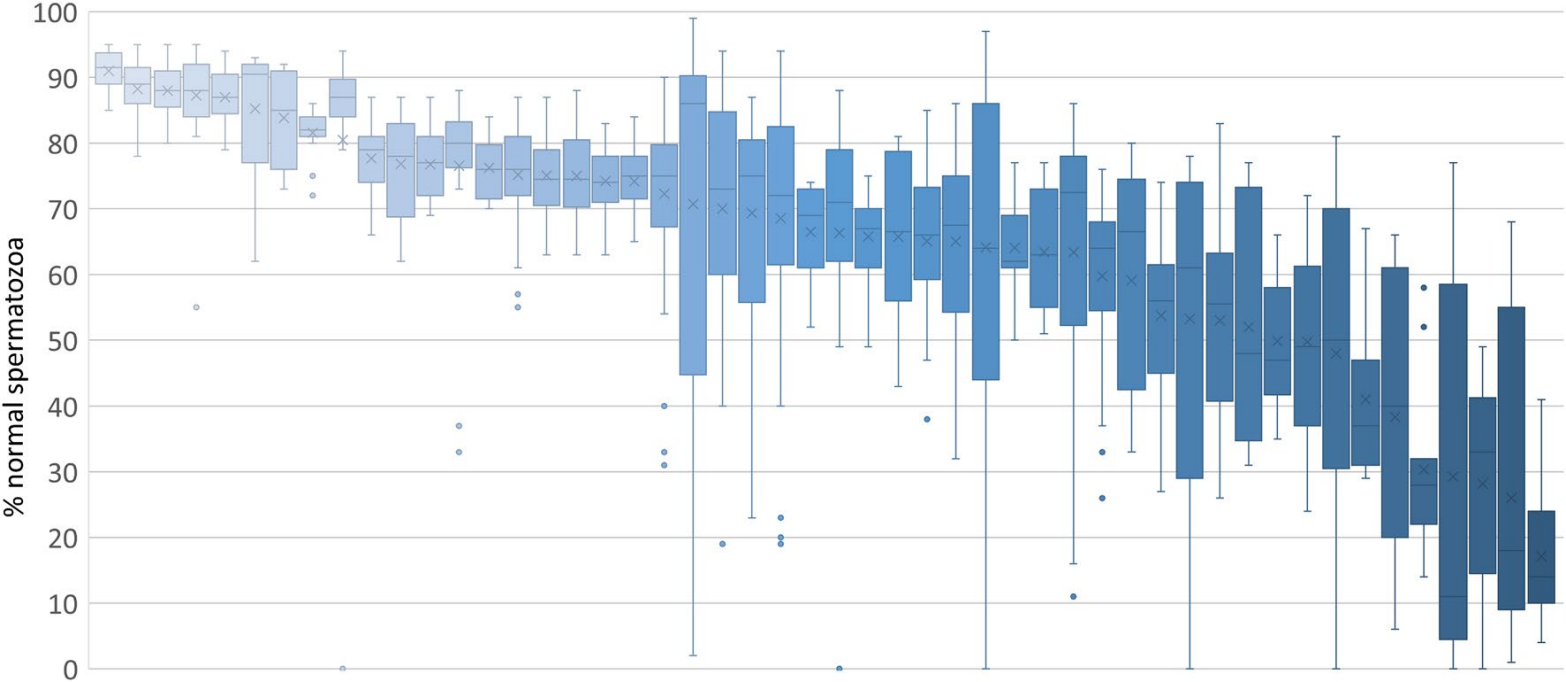
Changes in sperm normal forms. Semen samples were taken from bulls via electroejaculation and the percentage sperm normal forms were counted. The data show a box and whiskers plot consisting of 50 bulls, each of which had at least 5 different ejaculates across a minimum one month. Each box and whiskers plot represents an individual Bull showing the median, upper and lower quartile range. Outliers are represented by individual dots.

To understand variation in bull semen quality, we assessed 1271 ejaculates from 79 different bulls (11 different breeds) housed at Rockhampton stud farm, in the state of Queensland, Australia, over a period of 5 years (2014–2018). The raw data, together with the semen analysis and when the samples for each individual bull were collected is available in Supplementary 1. The climate in this area (23.3786° S, 150.5089° E) is considered sub-tropical, ranging from 16 °C in winter to over 30 °C in summer. A comprehensive semen analysis was undertaken, including sperm morphology and motility. To determine the variation in semen quality, we plotted the percentage of sperm normal forms for each bull that had 5 or more ejaculates taken annually. Morphology was used as a measure of sperm quality, as Söderquist et al.^17^ demonstrated that sperm motility is heavily influenced by the collection/collectors and, therefore potentially unreliable and irreproducible. This resulted in the analysis of 1178 ejaculates from 50 bulls, with an average of 23 ejaculates per bull. The percentage of sperm normal forms as a box and whiskers plot for each bull is given (Fig. 1). As shown, many bulls demonstrated extremely high variation between ejaculates, with several males ranging from < 50% normal forms (considered an outright “fail” in terms of cryopreservation potential) to > 70% (considered an outright “pass” in terms of cryopreservation potential) of normal sperm morphology. On the contrary, some bulls appeared to produce consistent semen samples across the year.

To determine the amplitude and the proportion of bulls demonstrating variation in the number of normal sperm forms, we measured the difference between the maximum and the minimum values recorded for each animal. From this analysis we found that: 9 (18%) bulls showed less than 20% variation in normal forms; 15 (30%) bulls had between 20–40% variation; 13 (26%) bulls were between 40–60% and for 13 (26%) bulls this number was over 60%. These data have major implications when interpreting semen analysis, since a bull could be classified as either fertile or infertile depending on which ejaculate was considered. This data also sheds light into why correlations between the vBBSE parameters such as morphology and the bull fertility are so variable.

### Seasonal effect on semen quality

Several sources of environmental influence have been suggested to affect bull sperm quality. These include feed availability (i.e., higher conception rates in rainy seasons)^27^, excessive protein intake^28^, day length^29^, thermal heat stress and age^30,31^. To better understand the dynamics of semen quality variation within our samples, we plotted sample “pass” and “fail” cryopreservation criteria against the month of collection. A raw bull semen sample is classified as “pass” when motility is above 60% and normal forms greater than 70%. When samples were between 30 and 60% motility and 50–70% normal forms, they were classified as a “compensatory” (or qualified) pass (q-pass). The compensatory pass relied on there being the ability to have at least 10 million motile normal forms of spermatozoa in each straw to allow for conception. An outright failure was given to any sample with less than 30% progressive motility or 50% normal forms. This allowed each ejaculate to be placed into a binary “pass” or “fail”.

**Figure 2:**
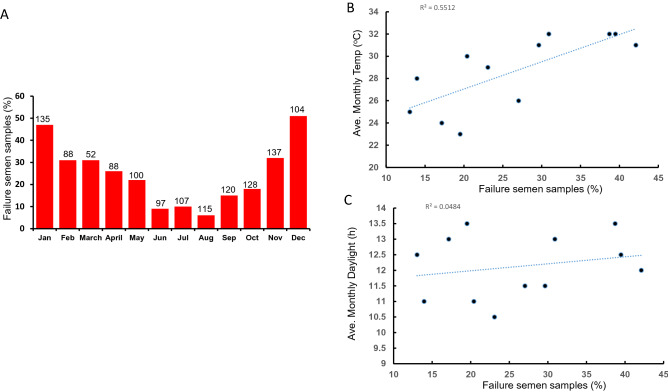
Seasonal variation in the semen quality of 1271 bull semen ejaculates. Semen samples were taken from bulls via electroejaculation and a full semen analysis was undertaken. Each sample was then classified as a pass or fail as described in Materials and Methods. (**A)** The percentage failure rate for each month is shown for all bulls. The number above each column indicate how many semen ejaculates were processed that month. (**B).** Scatter plot showing the average monthly temperature of Rockhampton and the percentage of samples that fail/month. Line of best fit indicates and r^2^ = 0.55. (**C)** Scatter plot showing the average daily sunlight in Rockhampton and the percentage of samples that fail/month. Line of best fit indicates and r^2^ = 0.04.

The data for the percentage of total males that “failed” within each month (1271 ejaculates) is shown (Fig. 2A). Clearly, there is a seasonal pattern, with over 90% pass rate in winter (June–August) that fell to 50% or lower in summer (Dec-Feb). Considering that all bulls were greater than 4 years old, housed on the same stud farm and received the same dietary supplement we found no relationship in terms of “pass” or “fail” rates to these parameters. Thus, the data clearly suggested that Temperature/Temperature-Humidity or day length were responsible for the increased failure rates seen during Summer. Therefore, to understand if there was any causal relationship, we correlated either the average monthly temperature (Fig. 2B) or daylight (Fig. 2C) with monthly failure rates. The data showed a correlation with monthly temperature (r^2^ = 0.55; and temperature-humidity index – see further modelling below) but not with daylight hours (r^2^ = 0.05). Combined, these data suggest that temperature was the most likely reason for increased failure rates during the warm/hot months.

### Changes in normal sperm forms categorised by breed

**Figure 3:**
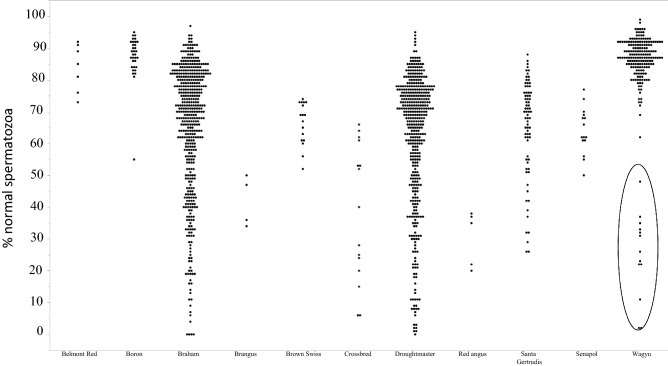
Variation in Semen quality as judged by Bull breed. Semen sample was collected and analysed for sperm morphology. The animals were then separated according to breed and the percentage normal forms for each ejaculate are shown.

The present study investigated 11 different breeds of cattle, and we reasoned that maybe one, or more breed(s) contributed to failure rates more than others. Therefore, we plotted the percentage of normal forms for every ejaculate against the breed (Fig. 3). All breeds showed similar variation except for the Belmont Red, Boran and Wagyu. However, a relatively small number of bulls from the Belmont Red and Boran breeds were assessed in this study, therefore, it is unclear if they are indeed more resistant to heat. In the case of the Wagyu, it is worth mentioning that only one animal exhibited poor sperm morphology in several ejaculates (Fig. 3 circled) during winter. A close inspection of the records showed that during this time the animal had a fever episode, with body temperature reaching 39.4 °C, and that the sperm morphology returned to normal in approximately 70 days.

### Some bulls are heat-sensitive, whilst others are heat-tolerant

**Figure 4:**
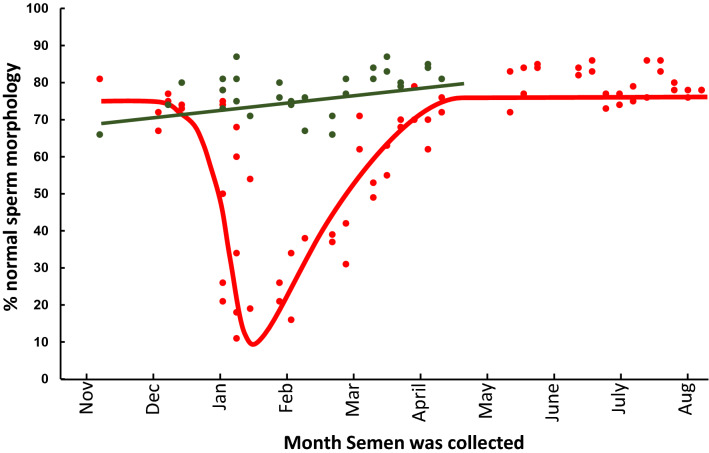
Identification of Heat-Sensitive and Heat-Tolerant bulls. The percentage normal sperm morphology from two bulls, both Droughtmasters, which had several ejaculates taken over the course of the year were plotted against the month in which the semen sample was taken. The first bull (red) is an example of a heat-sensitive bull. The second bull (Green) an example of heat-tolerant response.

Analysis of the present data clearly illustrated that some bulls showed marked variation in terms of their semen quality throughout the year (Fig. 1). Meanwhile, others demonstrated much less variation, and were reasonably consistent. To further clarify these differences, we closely analysed the percentage of sperm morphology from two bulls, both of whom had several ejaculates were taken throughout the year, including during and after summer (Fig. 4). There was a clear pattern, and evidence of two types of bulls. Prior to the summer season, bull 1 (Fig. 4, red), designated here as “heat-sensitive”, exhibited > 70% normal forms of spermatozoa. This value decreases dramatically, reaching its lowest point (10%) mid-January, before undergoing a recovery by April (> 70%). In contrast, bull 2 (Fig. 4, green) showed a consistent semen profile throughout the year. The data suggest this bull was more “heat-tolerant”.

**Figure 5:**
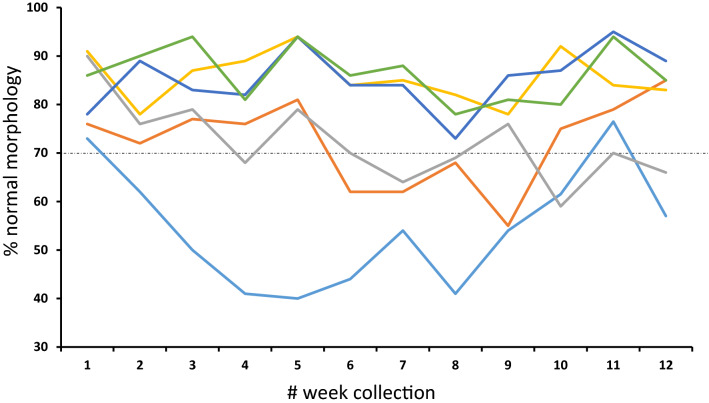
Heating of Wagyu bulls to identify heat-sensitive and heat-tolerant effect. Twenty Wagyu bulls all 3 years of age and over were heated to 40 °C for 12 h in an insulated barn. Before heating, bassline samples were taken (week 1). After heating, electroejaculation was used to collect semen every week for 11 weeks. For every sample, sperm morphology was counted by a qualified theriogenologist. The data show the percentage normal morphology for 5 bulls. The light blue line indicates a heat-sensitive bulls, whose morphology was affected by heat, then returned back to baseline. The orange and grey line represent two related bulls (same father) who also produced less than 70% normal forms. The yellow, green and dark blue lines represent three heat-tolerant bulls, whose semen profile did not drop below the 70% normal spermatozoa threshold.

To further explore the concept of “heat-tolerant” and “heat-sensitive” bulls, we subjected 20 Wagyu bulls to a single event of controlled heat stress (40 °C, 12 h). This experiment was performed during Winter, at Singleton (New South Wales, Australia, 32.5695° S, 151.1788° E), where the average temperature was 17 °C and never exceeded 18 °C. Prior to the heat stress event, baseline semen samples were taken from each animal. After heat stress, semen samples were taken every week for 11 weeks. During the experiment, two bulls were removed from the program due to infection and sickness whilst a 3rd bull was removed as it refused to co-operate with electroejaculation procedure. From the remaining bulls, we were able to reproduce the heat-sensitive and heat-tolerant bull phenomenon. The raw data from this work is given in Supplementary 1, and an example of the data is shown (Fig. 5). For 14 bulls, we found no difference in terms of their baseline samples, which were between 70–90% normal forms. This is consistent with the Wagyu bull characteristics and their heat-tolerance (Fig. 5, yellow, green, blue lines). Within these “heat-tolerant” bulls, there was a variation of 16–22% sperm normal forms. For the other three bulls, two of them showed a decline in sperm quality, which began 2–3 weeks after the heat event, dropping from a baseline of 85% and 90% normal forms to 55% and 59%, respectively (30–31% variation in normal forms; Fig. 5, grey and orange line). The third bull showed a greater degree of heat-sensitivity. Starting at 77% morphologically normal sperm, the spermiogram of this bull illustrated a rapid decrease in normal forms in a short time (2 weeks), reaching around 40% after 4–5 weeks. Sperm morphology remained at this level (37% variation in normal form) for four weeks, before recovery. These data show that under experimental condition, the phenomenon of heat-sensitive and heat-tolerant animals can be reproduced. Further, it appears that there are degrees of heat-sensitivity.

### Environmental heat stress leads to poor sperm quality 17 days later

Similar to previous reports, we noted that sperm quality does not begin to deteriorate until 2–3 weeks after the heat stress event of the bulls^32^. Based on the timing of spermatogenesis, this is consistent with reports that meiotic cells are more susceptible to heat stress following a heating event, with poor quality spermatozoa appearing in the ejaculate around 2–3 weeks later. To better understand the relationship between a “heat-event” and the production of poor-quality spermatozoa, we modelled both maximum temperature and maximum temperature humidity index (THI) and their relationship to the proportion of morphologically normal spermatozoa. The THI is an index representing the effect of humidity on the heat stress of an animal. THI was obtained using the following formula:

$$\mathrm{THI}=0.8* \frac{{T}_{max}}{100}+\frac{\left(humidity*\left({t}_{max}-14.4\right)\right)}{1}+46.4$$

where T_max_ = maximum temperature, (^o^F), and H = relative humidity.

**Figure 6:**
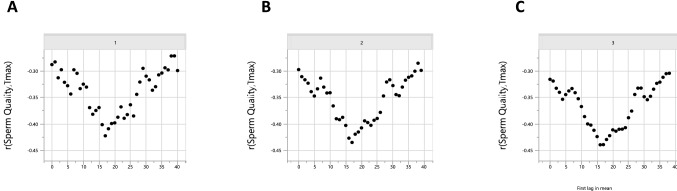
Bull semen quality (as percentage sperm normal forms) is related to the temperature that occurred 17–19 days ago. Correlation between sperm quality and maximum Temperature (Tmax). The Y axis is the Pearson correlation coefficient and X axis represents the number of days before the day the sperm sample was taken. (**a**) Uses one day of Tmax data whilst (**b**) averages two and (**c**) averages three consecutive days of Tmax data. The arrow shows the best correlation between Tmax and poor sperm quality, which occurs around 17–19 days before the semen sample is collected.

We plotted the correlation between semen quality and _Tmax_ on the day, and every day prior (up to 40 days) to semen collection (Fig. 6). This modelling demonstrated that poor semen quality was due to maximum daytime temperature 17 days prior (Fig. 6a, arrow). Notably, 1 day of heat-stress appears to be sufficient to cause poor sperm quality, since if we take the average of 2 (Fig. 6b) or 3-day maximal temperatures prior to collection (Fig. 6c) the correlation patterns were similar. Supplementary 3 shows further modelling for T_max_ and THI using between 1 and 5-day average temperatures prior to semen collection.

### Understanding the temperatures at which heat-sensitive bulls fail

**Figure 7:**
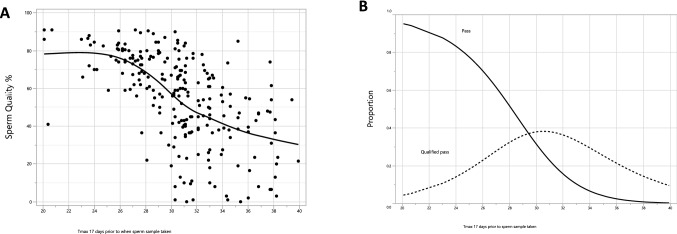
Sperm quality as a function of temperature 17 days prior. Sperm quality was evaluated for a subset of 12 heat-sensitive bulls in two ways, (**a**) quality as % for each sample taken (**b**) fitted lines from a logistic regression model estimating the proportion passing for two categories of sperm quality, a pass (quality 70% of better) and a qualified pass (quality 50% or better if pass failed).

To determine the T_max_ at which bulls in the paddock begin to produce poor quality spermatozoa, we modelled data using both parameters measured at 17 days prior to the heat event, and plotted samples from 12 heat-sensitive bulls (6 Brahmans, 4 Drought Masters and 2 Santa Gertrudis). The relationship between sperm morphology and T_max_ 17 days prior to heat even was plotted, with a spline smoothing cure to show the mean quality as a function of T_max_ (Fig. 7a). As the temperature increase, so the quality of sperm morphology decreases as expected. To gain further clarity, we next fitted a nominal logistic regression analysis to model the proportion of spermatozoa that would either pass, Q-pass or fail sperm cryopreservation criteria as a function of T_max_ 17 days prior. T_max_ effect was highly significant for both outcome categories, with both p < 0.001. The predicted proportions from the model for each pass condition at varying T_max_ were plotted (Fig. 7B). At lower temperatures (< 22 °C), the proportion of samples that would be expected to pass (0.90, solid line) or qualified-pass (Q-pass) (0.08, dotted line) summates to 0.98. This is similar to the actual number of samples that pass during winter (Fig. 2). However, as temperatures rise, the number of samples designated as “pass” begins to drop, while the number of qualified-pass samples increase up to 30.5 °C. These data show that a “q-pass” is likely reflecting the beginnings of the effect of heat stress. At this point, the proportion of samples with a q-pass has reached 0.38, whilst the proportion of samples with a direct “pass” has dropped to 0.22, giving and overall, 0.6 probable pass rate. At 34 °C, the proportion with an overall pass (pass 0.07 + q-pass 0.30) drops to 0.37 and continues to drop as temperatures rise above 34 °C. From a practical perspective, this data show that for heat-sensitive bulls, with temperature histories 17 days earlier of around 31 °C, 40% of the sperm samples collected will fail a spermiogram assessment and once the temperature exceeds 34 °C the failure rate will exceed 63%. As such, these data suggest that within heat-sensitive bulls, there are clearly some bulls that are more sensitive than others.

### Climate change modelling shows bulls are subjected to more heat events per decade

**Figure 8:**
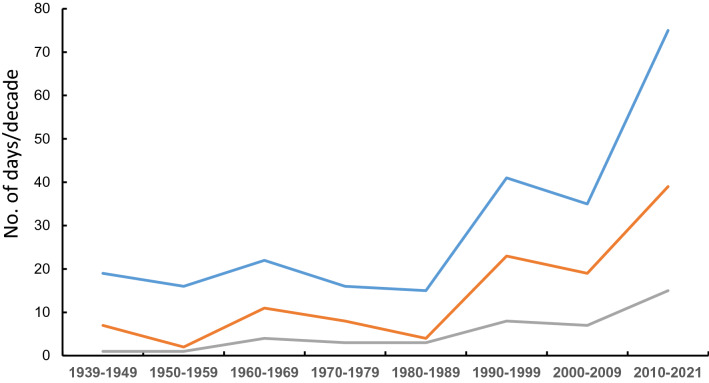
Climate change in Rockhampton from1939-2021. The historical record maximum temperature from Rockhampton weather station (23.38oS, 150.51oE), was downloaded which included data from 1939–2021 from bom.gov.au. The number of days per decade over 38 °C (blue),39 °C (orange)and 40 °C (grey) are shown.

To understand the impact of global warming, we downloaded historical temperature data from the Bureau of Meteorology using data from the Rockhampton site (bom.gov.au). We then counted the number of days/decade the maximum temperature exceed 32 °C–45 °C respectively. These data are shown in Supplementary Table 3. To give an example of “extreme” climate that would likely impact all heat-sensitive bulls, we plotted the number days/decade which were higher than 38 °C, 39 °C and 40 °C (Fig. 8) from the area in which the bulls were housed. As shown, the number of days per decade over these extreme temperatures jumps from 19, 7 and 1 (1939–1949; 38 °C, 39 °C and 40 °C respectively) to 75, 39 and 15 in the last decade. These data show the urgent need to identify heat-tolerant bulls as future sires.

## Discussion

In 1938, the first report of seasonal effects on breeding efficiency in dairy cattle was shown when the University of Nebraska dairy herd records demonstrated more services were required for conception during summer (May to October) from 1896 to 1934^33^. This same trend was also seen in records obtained from Purdue^34^ and Louisiana State University^35^ yet contradicted reports from Cornell University, whose records showed winter was the poorest season for breeding dairy cattle in Canada and New York State^36,37^. The underlying presumption was that the cow fertility was more thermos-sensitive than the bull fertility. It was not until 1941, when Anderson showed sperm volume and motility declined during summer in Kenya^36^ that thermo-sensitivity of bull fertility was established. Combined with experimental data on the heating of dairy bulls inside chambers (32.2 °C), there was evidence to show that a warm environment can lead to a decrease in sperm morphology and motility^38^.

Despite this data, controversy surrounding “season” and bull fertility continues to this day, with some reports showing no seasonal effect^13–17^, whilst others have improved semen quality in winter^16,17^ or summer^18,24^. However, inspection of that data shows the seasonal effect of heat stress can largely be explained by climate. Better sperm parameters in summer typically occurs where places experience sub zero temperatures in winter. Studies where season does not affect bull sperm quality are often in areas where temperature and humidity do not change substantially.

Our current work, which investigates predominately *Bos indicus* breeds of cattle used for meat purposes, shows that season definitely has an effect on bull semen quality. During winter (25 °C), samples had over 90% pass rate, whereas in summer (32 °C) this dropped below 50%. However, our work extends these observations and shows some animals more heat-sensitive than others. From our data, poor sperm production occurred in 63% of bulls once ambient temperatures exceeded 34 °C. Modelling of the temperature before the day of semen collections showed that poor quality spermatozoa do not appear until 17 days after the heat stress event which is consistent with our understanding of the heat-sensitive nature of meiotic (secondary spermatocytes) cells in the testis. However, for reaming ~ 40% of the animals, they seemed totally unaffected by 34 °C temperatures, producing good quality sperm all year round. In retrospect, our observation of heat-sensitive and heat-tolerant bulls may explain some of the previous data in experimental models. For example, in previous studies of six bulls that were subject to scrotal insulation, two showed large increases in abnormal spermatozoa (~ 60%), whereas others had fewer than 23% abnormal forms^39,40^. Furthermore, after 48 h scrotal insulation of 4 bulls, normal forms of spermatozoa had dropped to 0.5% in the first bull and 22% and 29% in bulls 2 and 3, respectively. However, the 4th bull, despite undergoing the same experimental insult, did not respond to thermal insulation, and maintained 82% normal forms for 3 weeks^41^. Thus, although not formally recognised nor commented on, it appears that heat-sensitive and heat-tolerant bulls have been previously demonstrated using other experimental models.

One potential confounder of our dataset was the mechanism by which semen was collected which in our case is via electroejaculation. Whilst the collection of semen from these animals does not take into account the libido of the animal, it does reflect the methods used by stud farms to generate semen straws for artificial insemination. Furthermore, when compared to the method of prostate massage, it appears the electroejaculation gives more consistent results^42^ in terms of semen quality.

From the data we analysed, the Wagyu breed stood out, in terms of heat-tolerance. Using temperatures of 40 °C (where according to our model, most bulls should fail), we managed to show remarkable heat-resistance in the Wagyu. In fact, several weeks post heat stress insult, only 3 of the 17 bulls started to show the typical signs of testicular heat stress, including poor sperm quality. One bull demonstrated a major change in normal forms, dropping from 70 to 40% over a period of five weeks. The other two bulls showed an intermediate drop from a baseline of 80% to 60% morphologically normal spermatozoa. Yet why is the Wagyu so tolerant? the answer may be genetic. Lineage tracing of the three animals that produced poor quality spermatozoa after heating showed they were half-brothers, same sire and different dams. Yet all the other animals were sired by a different bull. These data point to the possibility that heat-sensitivity could be inherited. This appears consistent in other model systems. For example, a “genetic” component to heat-sensitivity has been mentioned in one report where 1 genetic linage of Boars experienced a modest decrease in summer compared to two other lines^43^. In addition, heat-sensitive *Drosophila* males (i.e., produce no or low numbers of maggots after a heat event) sire other males that are also heat-sensitive^44^. Conversely, males that do produce maggots following the same heat event sire “heat-tolerant” male offspring^44^. In both cases, the female fly appears to have no control on whether the offspring are heat-sensitive or not. As such, thermo-sensitivity in *Drosophila* is thought to be passed on from sire to son through the Y-chromosome^44^.

The changing variation in semen quality, seen in roughly half of animals investigated here, sheds light into the predictive nature of the vBBSE. The ability of an animal to produce over 70% normal forms in one ejaculate, yet drop below 10% during summer, provides a clear explanation as to why the vBBSE can be a poor predictor of fertility. Additionally, identification of heat-sensitive bulls and removal from the herd/breeding pool would not only lift fertility rates but prevent the unnecessary culling of “empty” (non-pregnant) cows and overuse of bulls. Given the staggering rise in the number of days sub-tropical conditions are experiencing (Supplementary Table 2, Fig. 8) it is no wonder that these areas have reported historical lows in terms of fertility rates^45^. Identification of heat-tolerant bulls would add genetic gain, not only reducing the amount of animals needless used and culled, but as a consequence, reduce global methane emissions and lower food costs.

In summary, our data show that bulls respond to heat stress in a different manner, with some bulls being more prone than others. Given this, the future of the vBBSE and its correlation to fertility needs to be properly addressed, accounting for seasonal changes and individual responses to these changes. We suggest that the vBBSE be measured in animals in a window of 17–40 days following a heat event of over 36 °C. This will allow the detection of over 80% of bulls who’s semen profile is affected by heat, which should lead to better stock management and increased fertility rates in following generations.

## Supplementary Information

Supplementary Information 1.

Supplementary Information 2.

Supplementary Information 3.
