# Supplementary material for: Seasonal variation in bull semen quality demonstrates there are heat-sensitive and heat-tolerant bulls
Source: Sci Rep. 2022 Sep 12;12:15322. doi: 10.1038/s41598-022-17708-9 (PMC9468146; doi:10.1038/s41598-022-17708-9)
Supplement: Supplementary file 3 — Supplementary Information 3. [file 41598_2022_17708_MOESM3_ESM.pptx]

## Slide 1
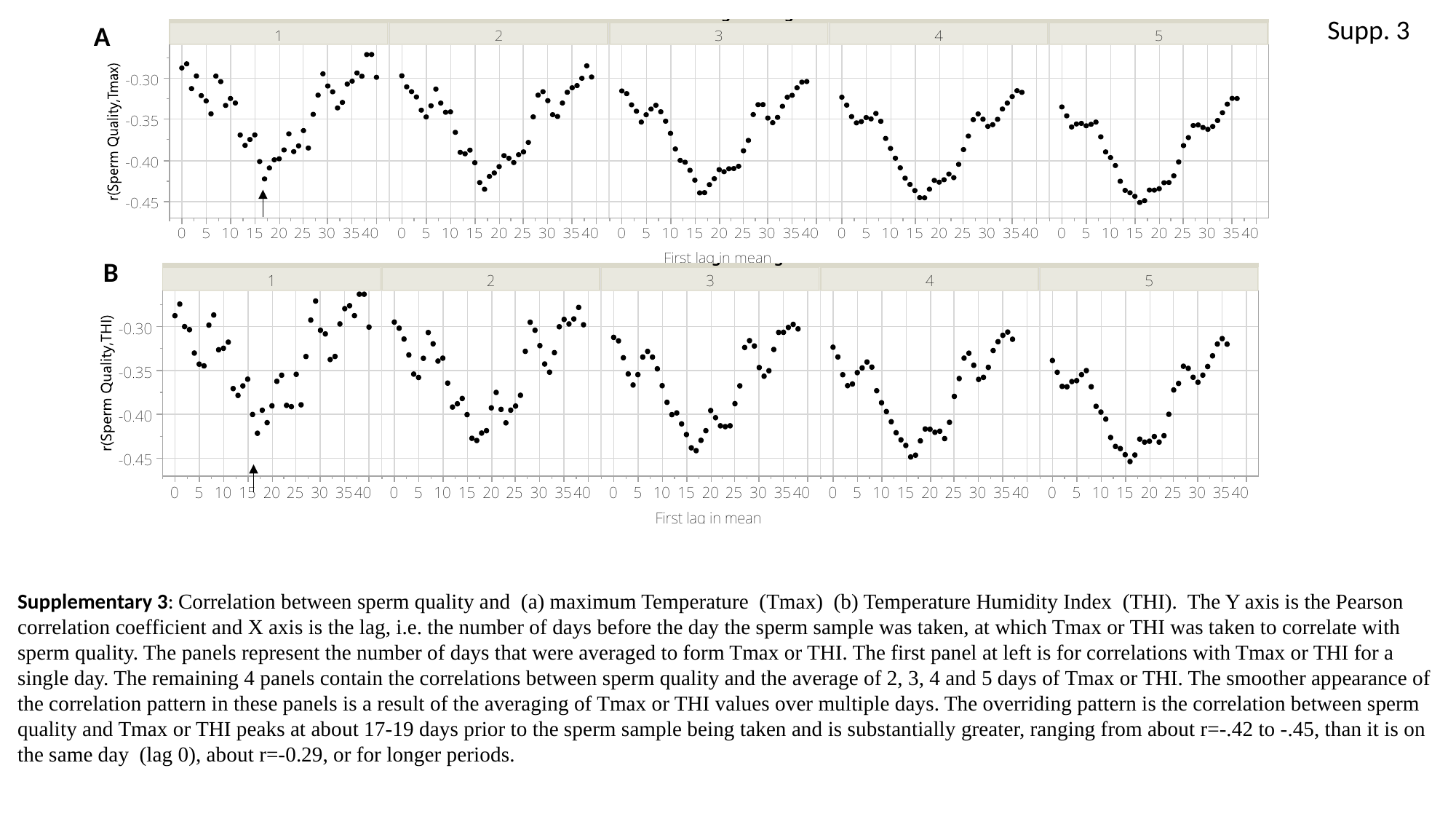

Supp. 3
A
B
Supplementary 3: Correlation between sperm quality and (a) maximum Temperature (Tmax) (b) Temperature Humidity Index (THI). The Y axis is the Pearson correlation coefficient and X axis is the lag, i.e. the number of days before the day the sperm sample was taken, at which Tmax or THI was taken to correlate with sperm quality. The panels represent the number of days that were averaged to form Tmax or THI. The first panel at left is for correlations with Tmax or THI for a single day. The remaining 4 panels contain the correlations between sperm quality and the average of 2, 3, 4 and 5 days of Tmax or THI. The smoother appearance of the correlation pattern in these panels is a result of the averaging of Tmax or THI values over multiple days. The overriding pattern is the correlation between sperm quality and Tmax or THI peaks at about 17-19 days prior to the sperm sample being taken and is substantially greater, ranging from about r=-.42 to -.45, than it is on the same day (lag 0), about r=-0.29, or for longer periods.
